# Editorial: Advances in Drug Formulation

**DOI:** 10.3389/fphar.2020.608771

**Published:** 2020-11-27

**Authors:** Biswajit Mukherjee, Johan Engblom, Paul Chi-Lui Ho, Veranja Karunaratne

**Affiliations:** ^1^ Department of Pharmaceutical Technology, Jadavpur University, Kolkata, India; ^2^ Department of Biomedical Science, Malmö University, Malmö, Sweden; ^3^ Department of Pharmacy, National University of Singapore, Singapore; ^4^ Department of Chemistry, University of Peradeniya, Peradeniya, Sri Lanka

**Keywords:** editorial, nanoliposomes, microwave ablation, nanoparticles, colon cancer, gastrointestinal tract

Advancement instils no ceiling. With vast amelioration of technology and blooming of biomedical information, a tremendous effort for improvement in drug delivery technology has been observed. The advancement of drug formulation has also been leading more toward further sophistication of drug transport systems that could deliver drug to site-specific cells/tissues/organs *in vivo*. Such target–specific drug delivery systems are needed because they can decrease dose and frequency of administration of drugs and thereby, reduce drug-related toxicity (Chakraborty et al., 2020). The present issue has highlighted several current areas related to the advancement of drug formulations.

In an interesting study, Wu et al. (2020) showed that doxorubicin-loaded liposomes enhanced the efficiency of electromagnetic waves (Microwave ablation, MWA) at solid tumors during hepatocellular carcinoma therapy. The investigators showed that doxorubicin-loaded nanoliposomes robustly enhanced mild MWA therapy in hepatocellular carcinoma, indicating their substantial tumor sensitizing ability. The researchers have claimed that the chemoablation therapy improved survival rates by enhancing the targeting efficiency. The formulations showed considerable promise for clinical HCC therapy.

Li et al. (2019) described the potentials of tocopherol polyethylene glycol 1,000 succinate (TPGS)/curcumin (CCM) micellar nanoparticles as a drug delivery system against colon cancer. The investigation showed that loading CCM into TPGS nanoparticles significantly enhanced its systemic absorption, increasing its Cmax, and producing a more sustained release profile, and increased apoptosis, and inhibited migration of HT-29 colon cancer cells. The nanoparticles efficiently released CCM in simulated colonic fluid and were significantly more efficacious than free CCM in reducing ROS concentration. Thus, the findings of the investigation shed light to test the nanoparticles against colon cancer in animal model in future study.

Moles et al. (2019) showed how malaria can be potentially managed with liposomal systems of diprotic basic drugs. The researchers have theorized novel distribution models to describe the partitioning behavior of diprotic basic drugs in 1,2-dioleoyl-sn-glycero-3-phosphocholine-based and red blood cell-analogous liposome suspensions. They further directed stable internalization of ionized drug microspecies into neutrally charged and anionic lipid bilayer leaflets in the form of ion pairs in association with anionic lipids. They have further claimed RBC as a clinically safe long-circulating carrier for polyprotic drugs, and the optimization of antimalarial therapies using RBC-targeted liposomal drug formulations.

Wang et al. (2019) showed that hepatic inflammatory proteins, CRP and TNF-α, and serum levels of IL1α were significantly increased in 5-PAHSA (palmitic acid esterified at the fifth carbon of hydroxy stearic acid)-treated db/db mice compared to the control mice. However, the effects of 5-PAHSA were not dose-dependent. Likewise, 5-PAHSA did not produce inflammatory factors in the experimental mice, suggesting chronic 5-PAHSA administration may induce systemic inflammatory responses. The researchers also showed that 5-PAHSA treatment improved glucose tolerance and insulin sensitivity in mice that received a high fat diet. But simultaneously, 5-PAHSA treatment showed no metabolic benefit in db/db mice. Thus, hyperglycemia condition in db/db mice may impair 5-PAHSA effects on metabolism. Finally, they postulated that the hyperglycemic conditions might impair 5-PAHSA action by inhibiting AMPKα mediated signals and promoting NF-κB-mediated inflammation.

Hua presented two interesting reviews and a mini-review article. In her first review Hua described the physiological and pharmaceutical considerations for rectal drug formulations (Hua (2019a)). Her profound scientific insights on the rectal route for drug delivery as a preferred route for drug administration with pronounced clinical or pharmaceutical perspectives are worth-mentioning. The rectal route may represent a practical alternative and drugs can be administered for both local and systemic action. The oral route of administration is the most preferred route by patients for gastrointestinal diseases. In her another review, she has provided a vivid understanding of the physiology of the gastrointestinal tract in healthy and diseased states and the use of it in drug delivery by modulating various physiological factors. Hua has opined for multiparticulate dosage systems to improve gastrointestinal drug delivery compared to single-unit dose common formulations (Hua, 2012). In her next erudite article (minireview), Hua showed her prudence describing significant advantages for the sublingual and buccal routes of systemic drug administration, substantiated by many current and focused research works in the areas (Hua, 2019b).

Many new thoughts and ideas along with their implementation have been enriching formulation development. Among the most recent approaches in the research of drug formulations, idea of nanorobots has emerged as a powerful therapeutic tool to develop highly efficacious precision medicines to localize the drug at the target site (da Silva Luz et al., 2016). The nanorobots have the ability to convert the minimum levels of physiological energy into propulsion and movement and can be steered to a target location under physiological environment and conditions leading to predominant improvement in therapeutic efficacy and reduction of systemic side-effects. They can also be programmed to release the therapeutic payloads with a precise release mechanism. Laser-driven nanoformulation for drug delivery is also another novel approach that needs attention.

In summary, drug-targets have already been preset in many diseases. Many sophisticated technologies, unique and powerful methodologies, and many novel pharmaceutical materials are available. Formulation scientists simply need to assemble them to develop smart and intelligent drug consignments that can accurately deliver the drug loads to preset targets. Successful development of many such accurate drug targeting-systems can bring a revolutionary change in drug formulations and human health care.

## Author Contributions

All authors listed have made a substantial, direct, and intellectual contribution to the work and approved it for publication.

## Conflict of Interest

The authors declare that the research was conducted in the absence of any commercial or financial relationships that could be construed as a potential conflict of interest.
